# The neuronal nucleus: a new battlefield in fight against neurodegeneration

**DOI:** 10.18632/aging.204519

**Published:** 2023-02-18

**Authors:** Eftekhar Eftekharpour

**Affiliations:** 1 Department of Physiology and Pathophysiology, University of Manitoba, Winnipeg, Manitoba, Canada

**Keywords:** neurodegeneration, nuclear lamina, epigenetics, retrovirus, lamin-B1

## Abstract

Aging is an inevitable fact of life which brings along a series of age-associated diseases. Although medical innovations and patient care improvement have increased our life expectancy, the rate of age-associated diseases have also increased. Nervous system is specifically prone to these diseases that cause neuronal loss in different anatomical regions. Alzheimer’s disease is the best-known example of age-associated illnesses and is diagnosed by accumulation of intracellular Neurofibrillary tangles and extracellular Amyloid Plaques resulting in dementia. However, therapeutic attempts aiming at the removal of these plaques and tangles to reverse the cognitive decline have generally failed in human patients and may compromise the patient’s health. We have learnt that interruption of neuronal housekeeping systems such as autophagy contributes to formation of these aggregates, and therefore understanding the underlying mechanisms that lead to failure of these endogenous protective systems may provide valuable information and novel therapies. The house keeping systems are delicately regulated through gene expression and chromatin modifications in the nucleus, however, the contribution of this largest cellular organelle in pathophysiology of the disease has been overlooked. During the last few years, a wealth of information on neuronal nucleus has emerged that provides a strong rationale for examining its contribution to the pathophysiology of the disease. In this research perspective, I have attempted to summarize the latest research on neuronal nucleus, with a special focus on nuclear lamina damage and its downstream events to rationalize the need for focusing on the neuronal nucleus as a therapeutic target.

## INTRODUCTION

Despite many years of research and clinical trials for neurodegenerative disorders, the cause of progressive loss of neurons in these devastating diseases remains controversial. Neurodegenerative disorders, including Alzheimer’s disease (AD) are on track of becoming a major global health crisis. The complexity of these disorders is best evidenced by the obvious challenges in finding effective treatments against its classic pathology markers; the insoluble amyloid aggregates in senile plaques and the hyperphosphorylated Tau-containing neurofibrillary tangles. Immunotherapies for removal of Amyloid Beta plaques are currently the forefront of treatments against the disease, although serious complications have been reported as development of Amyloid-Related Imaging Abnormalities (ARIA) such as ARIA-E (edema) or ARIA-H (hemorrhage) in AD patients (Sperling et al., 2012). Despite these controversies, accelerated approval of Aduhelm in 2021 is an indication of high demand for new treatments, even though its effectiveness remains to be proven in clinics (https://www.fda.gov/drugs/postmarket-drug-safety-information-patients-and-providers/aducanumab-marketed-aduhelm-information). There is currently no information available on the potential reversal of cognitive loss in patients undergoing these interventions. The standard pharmacological treatments currently in use are aimed at treating the symptoms rather than preventing the actual disease. There are many ongoing or terminated clinical trials that have failed to provide the desired effect; however, these disappointing outcomes while frustrating for the patients and their families, may hint that the senile plaques and tangles are not the cause of the disease, but they might be the consequence of unknown upstream events.

An increasing body of literature indicate changes in neuronal nuclei that are associated with neurodegeneration. These include structural damages to the nucleus accompanied by changes in chromatin rearrangement and robust changes in gene expression and even interruption of vital functions such as nuclear-cytoplasmic transport of RNAs and proteins. Whether targeting these events may lead to identification of novel disease modifying treatments, remains unknown. Over the last few years, few labs have focused on structural and molecular events that occur in neuronal nuclei. In this mini-review I have summarized the latest discoveries on the nuclear changes related to the field of neurodegeneration. I specially have highlighted the pioneering reports from Dr. Bess Frost (Barshop Institute for Longevity and Aging Studies) with those from my own research group. These reports despite being from different angles and using different specimens, show a mechanistic link between oxidative stress and nuclear damage in the context of neurodegeneration. These data cumulatively provide a strong rationale for the need for more exploratory research into nuclear involvement in neurodegeneration.

### Neuronal nucleopathy: an overlooked aspect of neurodegeneration

Research in the field of neurodegeneration has generally ignored the nucleus and instead has focused on neuronal cytoplasmic events. Evidence of nuclear involvement in AD has been shown by chromatin modifications and epigenetic changes that can be effectively inhibited by histone modifiers resulting in cognitive improvement in animal models [1]. The dynamic changes in neuronal nucleus and its response to various internal and external stressors in pathophysiology of AD have been recently reviewed [2]. The authors have detailed physical changes and stress-related responses including epigenetic changes that occur in neurons and will not be duplicated here. Changes in neuronal nucleus in AD also include interruption of mitochondrial-nuclear interaction through interruption of nuclear coded antioxidant proteins. Mitochondrial genome carries the genetic materials required for the oxidative phosphorylation machinery proteins, which are also the major source of ROS. The antioxidant machinery responsible for scavenging these ROS is provided by the nucleus. In neurons, high levels of ROS and mitochondrial activity are more important and must be closely regulated. In AD, decreased level of peroxisome proliferator–activated receptor γ coactivator 1α (PGC-1α) a master regulator of antioxidant proteins, impairs the functional interaction between mitochondria and nucleus [3]. Another implication of nucleus in pathophysiology of AD is attributed to changes in neuronal nuclear pore components. Nuclear pore complexes have a sophisticated structure and are the gatekeepers for transport of proteins and RNAs from nucleus to cytoplasm and vice versa. Although small proteins (<40kDa) can freely cross the pore, larger proteins rely on transport using a special group of GTP-binding nuclear protein also known as Ran-GTP. This has been shown to be interrupted after exposure to oxidative stress [4] or following accumulation of hyperphosphorylated tau in several neurodegenerative diseases including ALS, Huntington’s and AD. For a detailed review see Eftekharzadeh et al. [5]. While biochemical assays have extensively shown the involvement of nuclear envelope in regulation of chromatin compaction and gene regulation, a physical link between tau and nuclear damage in pathology of AD was originally reported by Feany’s group [6], by identifying coffee-bean shaped neuronal nuclei when stained for lamin-B1 protein. Understanding the upstream molecular mechanisms causing the nuclear lamina damage, and the downstream events that are involved in pathophysiology of AD may lead to identifying new treatment strategies.

### The nuclear lamina, structure, and function

The Nuclear Lamina (NL) is a protein-rich lattice at the interface of nuclear envelope and chromatin. Nuclear envelope is a continuation of endoplasmic reticulum on the cytoplasmic side, but it also continues inward and generates an intricate nucleoplasmic reticulum inside the nucleus in some eukaryotic cells including smooth muscle cells and cancer cells. It is composed of lamin A/C, lamin B1 and B2 (LB1/2) which bind to a vast array of lamin-binding proteins located in the inner nuclear envelope layer or on the chromatin. This strategic localization of NL provides a mechanical support for maintaining the nuclear shape and a regulatory gene expression for chromatin. Nuclear envelope is a highly dynamic structure and changes during cell cycle, cell death or in viral infections (reviewed here [7, 8]). The nuclear lamins and their binding proteins are closely regulated, and a combination of Unfolded Protein Response, Ubiquitin Protease System and Autophagy ensures their homeostasis [7].

Damage to NL, or laminopathy, is commonly caused by mutations in Lamin A protein, but due to its low levels in neurons, these diseases often spare the brain. Although there are some reports indicating the increased levels of lamin-A in late stages of Alzheimer’s disease [9], lamin-B1 is reportedly more important for neurons [10, 11]. The high dependency of adult neurons to lamin-B1 is attributed to its regulatory role for antioxidant gene expression, which is mediated by its direct interaction with Oct-1, a key transcription factor for antioxidant response genes [12]. Downregulation of LB1 has been reported in aging, which is associated with decreased levels of Oct-1, resulting in increased oxidative damage [12]. Lamin-B1 depletion has also been linked to decreased hippocampal neurogenesis in aging [13]. The importance of lamin-B1 is more highlighted due to its regulatory role for chromatin architecture, as it has been shown to be responsible for binding to heterochromatin, the silenced sequences in cellular genome. Downregulation of lamin-B1 or its receptor results in heterochromatin detachment from inner nuclear membrane and induction of cellular senescence [14]. Reorganization of neuronal chromatin may also be responsible for abnormal cell-cycle re-entry in neurons that is reported in AD as another cause of neuronal loss [15].

### Cause and consequence of nuclear damage

The pioneering discovery describing a structural link between tauopathy and NL damage was reported in a model of *Drosophila Melanogaster* [16]. Pan neuronal expression of a mutant form of human Tau in *Drosophila* was associated with chromatin relaxation and increased apoptosis. While disruption of heterochromatin formation was detrimental to neuronal health, enhancing heterochromatin formation increased neuronal survival. Frost and colleagues found that oxidative stress was also sufficient to drive loss heterochromatin’s decondensation [16], and that tau-induced over-stabilization of actin-caused nuclear envelope invagination, depletion of lamin-B1, and heterochromatin relaxation [6]. Using RNA sequencing in *Drosophila* the group showed that tauopathy increased nuclear transport of toxic RNA to the cytoplasm and within the nuclear invagination but was inhibited using pharmacologic or genetic interventions that inhibit RNA transport, which alleviated cell death. The group also showed the pathophysiological relevance of this mechanism in mouse [17] and in AD patient-derived brain tissue [18]. Promising results on application of anti-viral drugs and calorie restriction in *Drosophila* provided hope for the possibility of reversing the harmful effects of Tau through changing lifestyle or potential pharmacological interventions. The underlying mechanism for this abnormal gene expression pattern was shown to be due to downregulation of inherent repressive systems such as heterochromatin-mediated retrotransposon silencing and piwi-interacting RNA that are responsible for clearance of transposable elements [18].

Further studies from Frost’s group have expanded our current knowledge on the downstream nuclear signaling mechanisms linking tau to neurodegeneration. A toxic decrease in nuclear Ca^2+^ and downregulation of cAMP-response element binding protein and its downstream dependent genes, was observed in the *Drosophila* tauopathy model and induced pluripotent stem cells from patients with sporadic AD. Pharmacological activation of big potassium channels effectively elevated nuclear calcium levels and suppressed tau toxicity [19].

**Figure 1:**
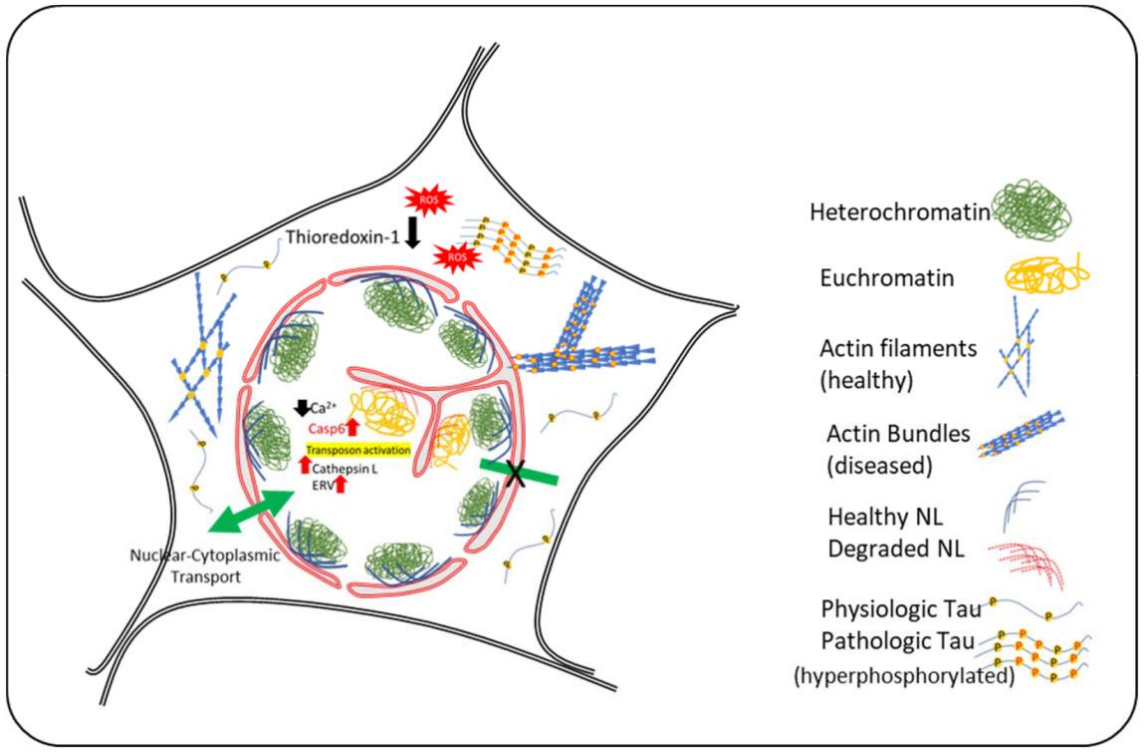
**Schematic Diagram summarizing the events in neuronal nuclear invagination in AD: Causes and consequences.** Oxidative stress and Tau-hyperphosphorylation have been shown to be responsible for nuclear envelope invagination in neurons. Abnormal actin bundling due to tau-hyperphosphorylation and increased oxidative stress result in changes in nuclear lamin and degradation of nuclear lamina proteins by caspase-6 and cathepsin L that are upregulated in AD. The molecular events downstream of nuclear invagination are mediated by changes in chromatin compaction and abnormal gene transcription, including activation of transposons and emergence of ancient retroviruses. Experimental evidence indicate that attenuation of these events may represent new therapeutical approaches.

The contribution of NL invagination in Tauopathy in Frost’s lab, has also been expanded to include the interruption of normal cellular activities that are especially important for maintaining neuronal health. Nuclear envelope invagination affects RNA export into the cytoplasm, as shown by accumulation of polyadenylated RNA within the nuclei and in proximity to the invaginated envelope. This is shown to be associated with neurodegeneration [20]. A question remains as to whether accumulation of these RNA is due to downregulation of RNA housekeeping systems such as nonsense-mediated RNA decay (NMD) in nuclei, or changes in nuclear pore transport. Although it was originally postulated that NMD is a regulatory mechanism aimed at degradation of mutated or pre-mature RNA, recent advances show its involvement in regulation of gene expression in normal conditions. Frost’s research showed that pharmacological approaches to increase NMD activity effectively suppressed tau toxicity [21]. Further research into tauopathy mechanisms in Frost’s lab showed a potential impact of Tau toxicity on decreased synaptic activity in *Drosophila*. They have recently reported that activity regulated cytoskeleton associated protein (ARC), which is upregulated in the brain of AD patients, is not effectively transported into the synaptic vesicles and presynaptic membrane and instead is accumulated in the nucleus and neuropil. Genetic reduction of *Drosophila* ARC protein was shown to be neuroprotective against tauopathy [22]. A summary of nuclear events is shown in Figure 1.

Although the reports from Frost’s lab provide a deep understanding into the cause and consequences of NL invagination and showed the potential interventions that can alleviate neurodegeneration, the underlying molecular mechanisms mediating NL invagination have not been adequately examined. My lab has focused on identification of protease systems that can cause lamin B1 degradation and NL invagination. We have shown that NL invagination is not unique to Tauopathy and reported that exposure of human neuroblastoma cells to serum deprivation may also result in mild NL damage [23]. Inhibition of Thioredoxin1 (Trx1) system in these conditions exacerbated NL damage and was associated with degradation of lamin B1. Trx1 is a major cellular redox protein responsible for maintaining many cellular proteins in their optimal physiologic activities. Trx1 has been best known for its anti-apoptosis activity that is mediated through its inhibitory effect for Apoptosis Signal Kinase-1 (Ask1) and Caspase-3 activation [24]. Of particular interest, Trx1 levels have decreased in the brains of AD patients [25]. Our group showed that Trx1 downregulation was associated with caspase-6 activation and its inhibition was prevented by caspase-6 inhibitor. Further examination of this system confirmed that only inhibition of Trx1, but not glutathione was able to mediate caspase-6 activation and NL damage. These results were also confirmed in the widely used 3xTg mouse model of AD [23]. Caspase-6 is also considered an early marker of AD that might contribute to neurodegeneration and memory deficit as shown in a transgenic mouse model overexpressing caspase-6 with memory impairment. Inhibition of caspase-6 in this model effectively inhibited the memory loss [26]. The depletion of Trx1 system was also sufficient to interrupt autophagy lysosomal in neural cells [27], which is also a prominent finding in AD pathophysiology.

A second protease enzyme was subsequently identified by my team [28]. Using the amyloid beta 42 toxicity (Aβ42) model in primary hippocampal and cortical neurons, as well as human neuroblastoma cells, we showed that cathepsin L is also capable of lamin B1 digestion, and this cleavage pattern is distinct from that of caspase-6. NL invagination was also evident in this model, indicating that NL invagination is not limited to tauopathy model. Aβ42 toxicity model also showed similar histone modifications to human post- mortem tissue (Islam et al., 2022). Interestingly, while in 3xTg mice, both unique patterns of NL cleavage by caspase-6 and cathepsin L were observed, the Aβ42 toxicity model and human brain tissue from AD patients only showed the cathepsin L mediated fragment. We hypothesized that the lack of caspase-6 mediated cleavage of lamin B1 in post-mortem AD tissues might be due to cathepsin L proteolytic degradation of caspase-6. Using a cell-free enzymatic assay the group showed that caspase-6 is in fact a substrate of cathepsin L [28].

The functional importance of two different proteolytic degradation for lamin by caspase-6 and cathepsin L is not clear; however, we proposed that caspase-6 might be an early player in induction of NL invagination following Trx1 depletion, while cathepsin L activation occurs in later stages of cell death in AD after induction of lysosomal permeability. Alternatively, the two different mechanisms may occur concurrently in different cell types, although this remains to be examined.

Overall, since the need for identification of new treatments for treatment of neurodegenerative diseases remains a high priority in biomedical research, the growing literature on nuclear involvement in pathophysiology of these diseases provides a strong rationale for including the nucleus as a therapeutic target. The long-lasting events associated with neurodegenerative diseases are indicative of disruption of ongoing homeostatic programs that are ultimately regulated by gene expression in the nucleus, and therefore understanding the upstream events leading to and following the nuclear events may hold the key to new therapies that can modify the disease’s course. It is now well documented that neurodegeneration starts many years before its definitive clinical diagnosis, and therefore until identification of biomarkers associated with the disease, non-pharmacological preventive approaches must be highly considered. Evidence of physical activity to induce neurogenesis in humans and calorie restrictions to limit nuclear laminopathy effects in *Drosophila* are good examples on how changing our lifestyle is needed and may be effective for decreasing the chance of developing diseases such as AD.
